# Cheoplastic Process

**Published:** 1857-07

**Authors:** 


*Cheoplastic Process.*—At the last meeting of the St. Louis Dental Association this new method was taken into consideration, and reported upon to the society by a very judicious committee, and we refer our readers to their report found in the first part of this No. for full particulars.

The results of the accumulated experience of those using this process since our last issue, fully justifies our own expressions of its usefulness and applicability, and from many letters now before us from leading men in the profession who use this method, we find the fullest expressions of gratification, particularly in that class of cases found to possess insurmountable obstacles to the old plan of swaged plate. We have letters recording successes in cases where the scattered condition of the teeth to be supplied have rendered the use of clamps indispensable in gold plates where this new process has been attended with the completest success. We, in consequence, more than ever entertain the opinion that *Cheoplasty* must go into universal use.

